# TLR9-Dependent and Independent Pathways Drive Activation of the Immune System by *Propionibacterium Acnes*

**DOI:** 10.1371/journal.pone.0039155

**Published:** 2012-06-22

**Authors:** Sandrine Tchaptchet, Marina Gumenscheimer, Christoph Kalis, Nikolaus Freudenberg, Christoph Hölscher, Carsten J. Kirschning, Marinus Lamers, Chris Galanos, Marina A. Freudenberg

**Affiliations:** 1 Department of Developmental Immunology, Max Planck Institute of Immunbiology und Epigenetics, Freiburg, Germany; 2 Institute of Pathology, University Medical Center Freiburg, Freiburg, Germany; 3 Division of Infection Immunology, Research Center Borstel, Borstel, Germany; 4 Institute of Medical Microbiology, University of Duisburg-Essen, Essen, Germany; Charité-University Medicine Berlin, Germany

## Abstract

*Propionibacterium acnes* is usually a relatively harmless commensal. However, under certain, poorly understood conditions it is implicated in the etiology of specific inflammatory diseases. In mice, *P. acnes* exhibits strong immunomodulatory activity leading to splenomegaly, intrahepatic granuloma formation, hypersensitivity to TLR ligands and endogenous cytokines, and enhanced resistance to infection. All these activities reach a maximum one week after *P. acnes* priming and require IFN-γ and TLR9. We report here the existence of a markedly delayed (1–2 weeks), but phenotypically similar TLR9-independent immunomodulatory response to *P. acnes*. This alternative immunomodulation is also IFN-γ dependent and requires functional MyD88. From our experiments, a role for MyD88 in the IFN-γ-mediated *P. acnes* effects seems unlikely and the participation of the known MyD88-dependent receptors, including TLR5, Unc93B-dependent TLRs, IL-1R and IL-18R in the development of the alternative response has been excluded. However, the crucial role of MyD88 can partly be attributed to TLR2 and TLR4 involvement. Either of these two TLRs, activated by bacteria and/or endogenously generated ligands, can fulfill the required function. Our findings hint at an innate immune sensitizing mechanism, which is potentially operative in both infectious and sterile inflammatory disorders.

## Introduction

The Gram-positive bacterium *Propionibacterium acnes* (formerly *C. parvum*) is a part of the human and mouse flora and an opportunistic human pathogen. It has been implicated as an etiologic agent of specific inflammatory diseases, including sarcoidosis [1]–[3]. Infection with live or administration of heat-killed *P. acnes* in experimental animals and humans leads to a strong activation of the reticulo-endothelial system. In mice, *P. acnes* induces several immunomodulatory activities, including intra-hepatic granuloma formation, splenomegaly, enhanced resistance to infection and malignant tumors as well as hypersensitivity to microbial Toll-like receptor (TLR) agonists, such as lipopolysaccharide (LPS), lipopeptides (LP) or synthetic CpG DNA analogs [4]. The hypersensitivity is characterized by an overproduction of pro-inflammatory cytokines and an enhanced lethality in response to TLR triggering. Concomitantly, *P. acnes*-primed mice develop hypersensitivity to the endogenous mediators TNF-α and IL-12 [4]–[6].

The development of *P. acnes* effects is dependent on an extremely slow degradation of internalized bacteria in macrophages. This leads to macrophage activation [7], [8]. The effects of *P. acnes* become detectable 3 days after priming and peak 4 days later, are dependent on *P. acnes* recognition *via* TLR9 in macrophages [6], [9] and require IL-12-mediated IFN-γ production [5], [10], [11]. IFN-γ, the actual mediator of sensitization, is produced mainly by CD4^+^ T cells and NK cells, however, other T cell subsets (CD8^+^, γδT cells) can also provide this cytokine, independently of their antigenic specificity. Interestingly, *P. acnes* specific T cells are not necessarily required. Thus, T cells play an important, antigen-independent role in the activation of the innate immune system by this intracellular microbe. Moreover, NK cells can substitute for T cells in the production of IFN-γ, however higher amounts of *P. acnes* have to be used for priming [6].

Earlier it was shown that only a degradation-resistant *P. acnes* strain exhibited immunostimulatory and anti-tumor activities in mice, while a degradation-sensitive strain was inactive [8]. In another study, the ability of *P. acnes* strains to activate the reticulo-endothelial system was associated with the resistance of the phagocytosed bacteria to degradation in macrophages [7], indicating a positive correlation between the immunostimulatory activity of *P. acnes* and its relative resistance to degradation.

In agreement, we show here in mice that *P. acnes* and its immune effects disappear concomitantly, i.e. between 4 to 6 weeks after intravenous administration of the bacteria. We show further that the absence of TLR9 slows down the bacterial degradation. Unexpectedly, we found that TLR9-deficient mice also develop the typical symptoms of hypersensitivity; however, their development is strongly delayed. Finally, the underlying mechanisms of TLR9-independent immune activation by *P. acnes* are addressed in this study.

## Results

### Prolonged Persistence of Bacteria and Delayed Granuloma Formation in the Liver of *P. acnes*-primed TLR9^−/−^ mice

**Figure 1:**
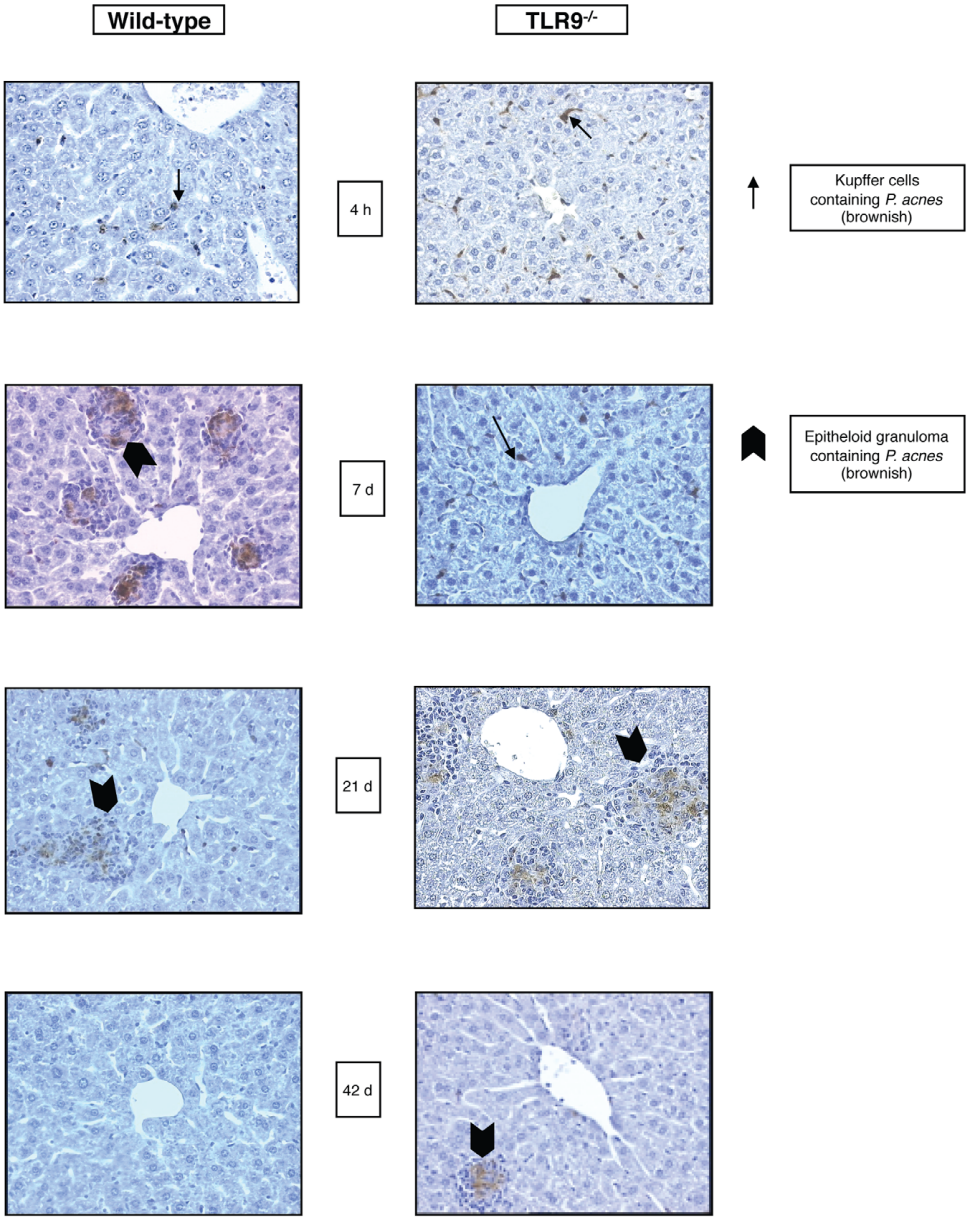
Persistence of *P. acnes* in livers of WT and TLR9^−/−^ mice. Groups of five WT and TLR9^−/−^ mice were treated with heat-killed *P. acnes* (100 µg/g b.w.) i.v. and livers were removed at different time points after treatment. Immunohisto-chemical staining of liver sections using *P. acnes* specific antiserum shows brown *P. acnes*-specific staining within granulocytes, sinusoidal lining cells and/or granulomas. Left panel: liver of WT mice*; right panel*: liver of TLR9^−/−^ mice. One representative slide per group of five animals is shown.

We showed previously that the absence of TLR9 impairs the development of immunomodulatory effects by *P. acnes* [9]. Here we investigated, whether the absence of TLR9 influences in any way the degradation of *P. acnes*. We injected heat-killed *P. acnes*, i.v. into wild-type (wt) and TLR9^−/−^ mice and examined the presence of bacteria in liver, spleen and kidney at different times (up to day 42 after priming) by immunohistochemistry. In wt and TLR9^−/−^ animals the bacteria, visualized by the characteristic brown staining, were present almost exclusively in the liver (Fig. 1), while spleen and lung remained bacteria-free for the duration of the experiment (not shown). Four hours after injection, *P. acnes* was detectable in the liver associated mainly with sinusoidal lining cells in both wt and TLR9^−/−^ mice.

In wt mice, from day 3 on after priming, intrahepatic granulomas consisting of mainly mononuclear, partially *P. acnes*-positive cells were observed. However, a portion of the *P. acnes* remained associated with the sinusoidal lining cells. The number and size of granulomas reached maximum between days 7 (Fig. 1, left) and 10 after priming, was moderately reduced after 21 days (Fig.1, left) and granulomas disappeared after 42 days (Fig 1, left). The disappearance of granulomas was accompanied by an almost complete loss of *P. acnes* detectability.

In TLR9^−/−^ mice *P. acnes* remained associated exclusively with the liver sinusoidal lining cells and no granuloma formation was observed in this organ during the first 7 days after administration. From day 10 on after priming, however, granulomas containing *P. acnes*-positive cells appeared in the liver. Their number reached a maximum on day 21 and these cells were still sporadically present 42 days after *P. acnes* administration (Fig. 1, right). Our results show a contribution of TLR9 to the elimination of *P. acnes* from the host and reveal the existence of a delayed, TLR9-independent mechanism of granuloma formation in response to *P. acnes* treatment.

### Delayed Development of Sensitization and Splenomegaly in *P. acnes*-treated TLR9^−/−^ mice

**Figure 2:**
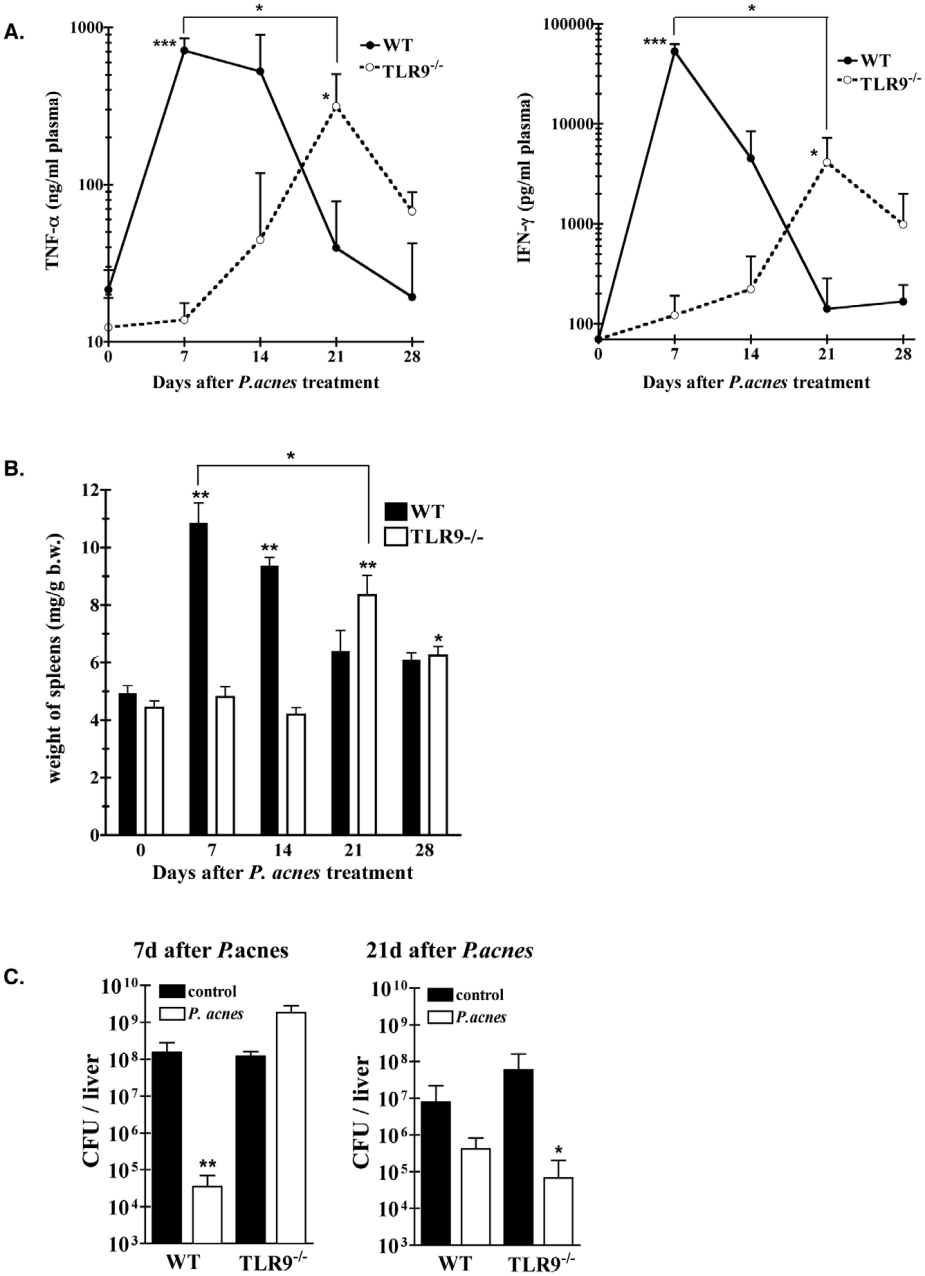
Delayed development of *P. acnes* effects in primed TLR9^−/−^ mice. Groups of WT and TLR9^−/−^ mice (15–20 animals/group) were primed with heat-killed *P. acnes* (20 µg/g b.w.) i.v. or remained untreated (controls; day 0). **A. Hypersensitivity to LPS:** At indicated time points after priming the animals were challenged with LPS from *S.a.e.* (0.01 µg/g b.w.), i.v. One hour and 4 h later, plasma was collected for determination of TNF-α and IFN-γ, respectively. Before challenge with LPS no detectable TNF-α or IFN-γ was found in plasma of *P. acnes*-treated mice of either one of the groups (not shown). Cumulative data of 3 different experiments are shown. *:p-value<0.05 and ***:p-value<0.001 WT compared to TLR9^−/−^ mice, and *P. acnes*-treated WT or *P. acnes*-treated TLR9^−/−^ at the indicated time point compared to untreated controls. **B. Splenomegaly:** Spleens were removed and weighted at indicated time points after treatment. Cumulative data from 4–5 experiments are shown. *:p-value<0.05 and **:p-value<0.01. **C. Enhanced resistance to serovar Typhimurium infection is delayed in TLR9^−/−^ mice:** 7 or 21 days after priming, the animals were infected with *Salmonella* serovar Typhimurium (200 CFU/0.2 ml PBS/i.v.). Bacterial counts in liver of unprimed and primed animals were determined 4 days after infection. One representative experiment of three is shown. *:p-value<0.05 and **:p-value<0.01.

The unexpected finding of delayed granuloma formation in primed TLR9^−/−^ mice prompted us to investigate whether other characteristic immunomodulatory effects of *P. acnes* also appear with a similar delay in such animals. For this purpose, we compared the LPS sensitivity and spleen weights of wt and TLR9^−/−^ mice at different time points (up to day 28) after *P. acnes* priming. Plasma levels of TNF-α and IFN-γ induced by an LPS challenge served as a measure of sensitization (Fig. 2A). In agreement with our earlier studies [9], in wt mice sensitization to LPS peaked by day 7 after priming, while in TLR9^−/−^ mice no sensitization was observed at this time point. Furthermore, the cytokine hyper-responses to LPS of primed wt mice decreased slightly by day 14 and considerably by day 21 and 28 after *P. acnes* administration. Enhanced TNF-α and IFN-γ responses to LPS also appeared in *P. acnes*-primed TLR9^−/−^ mice, but with a marked delay. They were first detectable on day 14 after treatment, peaked by day 21 and declined again, but were still slightly enhanced on day 28 after *P. acnes* administration (Fig. 2A). Thus, the development of LPS hypersensitivity in *P. acnes*-primed mice lacking TLR9 occurs, but it is delayed and of lower magnitude as in primed wt mice (Fig. 2A). As shown in fig. 2B, in both wt and TLR9^−/−^ mice the development of splenomegaly, another characteristic *P. acnes* immune effect, correlated timely with the development of *P. acnes*-induced LPS hypersensitivity. Thus, while the characteristic enlargement of the spleen peaked in wt mice on day 7 after priming, we observed in TLR9^−/−^ mice a delayed and less pronounced enlargement of the organ that reached maximum on day 21.

### Delayed Development of Enhanced Resistance to Typhoid Fever in *P. acnes*-treated TLR9^−/−^ mice

Another hallmark of *P. acnes* treatment is the development of an enhanced resistance to infection in the primed animals [12], [13]. In wt mice, peak values of this activity are reached 7 days after priming. Here we tested whether in the absence of TLR9 signaling this enhanced resistance to infection develops with a delay, like sensitization to LPS. For this purpose we infected wt and TLR9^−/−^ mice on day 7 or 21 after priming, as well as the respective unprimed controls with 200 CFU of *Salmonella* serovar Typhimurium and determined the number of viable bacteria in the liver 4 days later. As shown in Fig. 2C and in agreement with our earlier study [9], on day 7 after priming only wt, but not TLR9^−/−^ mice were resistant to *Salmonella* infection, i.e. exhibited significantly lower numbers of live bacteria in the liver compared to the unprimed controls. However, on day 21 after *P. acnes* priming we observed significantly enhanced resistance to *Salmonella* serovar Typhimurium in TLR9^−/−^ mice, while in wt mice the resistance to infection returned to the pre-priming level. The above data show that the absence of TLR9 delays the development of enhanced resistance to infection, mirroring the development of other immunomodulatory *P. acnes* effects shown above.

### Requirement for IFN-γ and IL-12 in both Early and Late Immune Activation

Mice lacking functional IFN-γ or IL-12 do not develop the characteristic immune effects up to day 7 after *P. acnes* treatment [14]–[16]. This is confirmed in the present study using IFN-γR^−/−^ and IL-12Rβ2^−/−^ mice (Figure S1 and not shown). We tested further whether the delayed development of sensitization in *P. acnes*-treated mice also requires intact IL-12 and IFN-γ signaling. As shown in Figure S1, both IFN-γR^−/−^ and IL-12Rβ2^−/−^ mice failed to exhibit LPS hypersensitivity also on day 21 after *P. acnes* treatment. Furthermore, splenomegaly and intrahepatic granuloma formation were always absent from these mice (not shown). Thus, both IL-12 and IFN-γ are involved in the immunomodulatory response to *P. acnes* priming.

### Complete Absence of *P. acnes* Effects in MyD88-deficient mice

**Figure 3:**
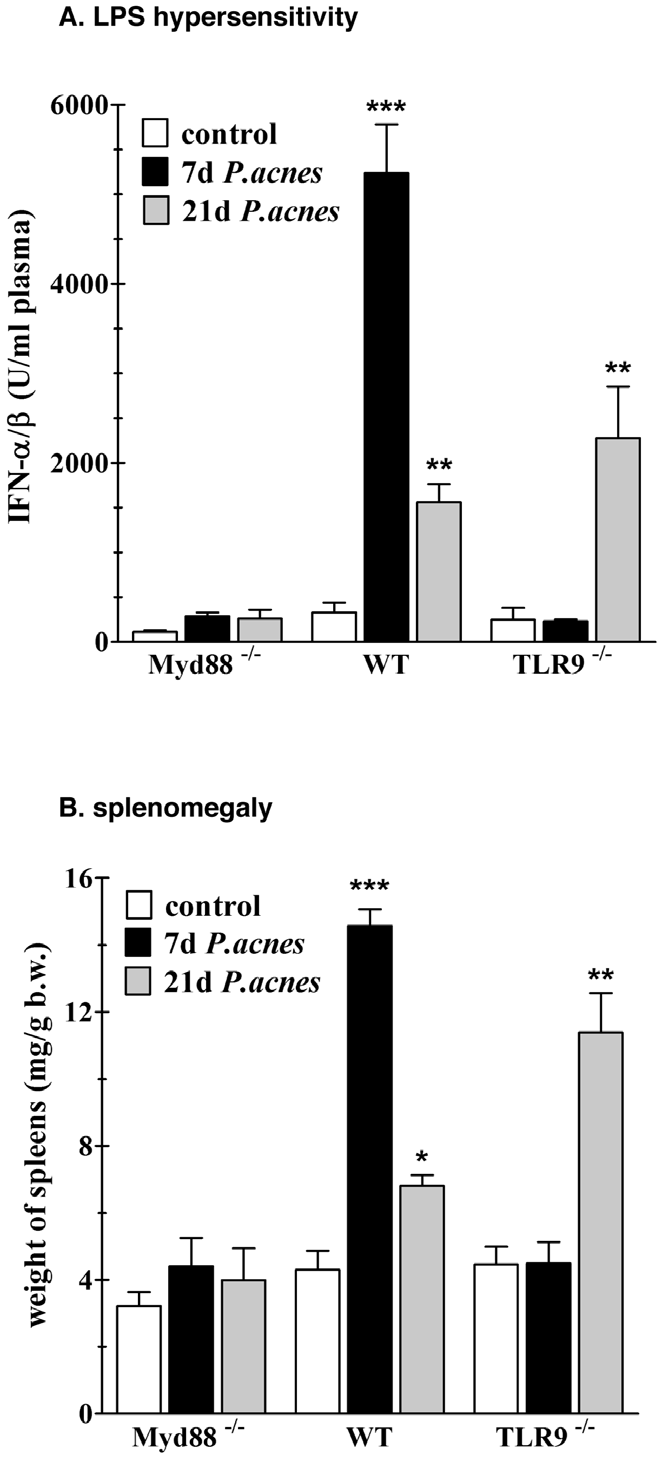
Absence of hypersensitivity to LPS and splenomegaly in MyD88^−/−^ mice after *P. acnes* treatment. Groups of four to five WT, TLR9^−/−^ and MyD88^−/−^ mice were treated with heat-killed *P. acnes* (20 µg/g b.w.) i.v. or remained untreated (control). 7 and 21 days after priming the animals were challenged with LPS *S.a.e.* (0.01 µg/g b.w.) i.v. and plasma was collected two hours later for determination of IFN-αβ (**A**). The spleens were removed and weighted (**B**). One representative experiment of many is shown. *:p-value<0.05, **:p-value<0.01 and ***:p-value<0.001.

The delayed development of the characteristic *P. acnes* effects in TLR9^−/−^ mice raised the question whether another TLR may mediate the delayed effects in the absence of TLR9. Since MyD88 is an adaptor essential for signaling through most of the TLRs [17], we compared the development of *P. acnes* effects in MyD88^−/−^ mice to that in wt and TLR9^−/−^ mice on day 7 and 21 post priming. We tested for the occurrence of splenomegaly and intrahepatic granuloma formation, and determined the MyD88-independent IFN-αβ response as a measure of LPS sensitivity. As shown in Fig. 3A, wt mice exhibited the expected strong IFN-αβ hyper-responses to LPS challenge on day 7 and weaker, but still significant, on day 21 after priming, while TLR9^−/−^ mice exhibited only the late LPS hypersensitivity on day 21. On the contrary, MyD88^−/−^ mice did not develop LPS hypersensitivity at all. Furthermore, unlike the other strains of mouse, primed MyD88^−/−^ animals developed neither splenomegaly (Fig. 3B), nor the typical intrahepatic granulomas (not shown). Thus, the absence of MyD88 abolishes both, the early and the late development of *P. acnes* effects. The requirement for MyD88 during the early sensitization could be explained easily by the requirement for TLR9. However, Sun and colleagues reported that MyD88^−/−^ mice exhibit impaired responses to IFN-γ [18]. Since IFN-γ is a key mediator of the early and late immunomodulatory effects of *P. acnes,* we first investigated whether a malfunction of IFN-γ might be responsible for the absence of sensitization in MyD88^−/−^ mice.

### IFN-γ-induced LPS Hypersensitivity in MyD88^−/−^ mice

**Figure 4:**
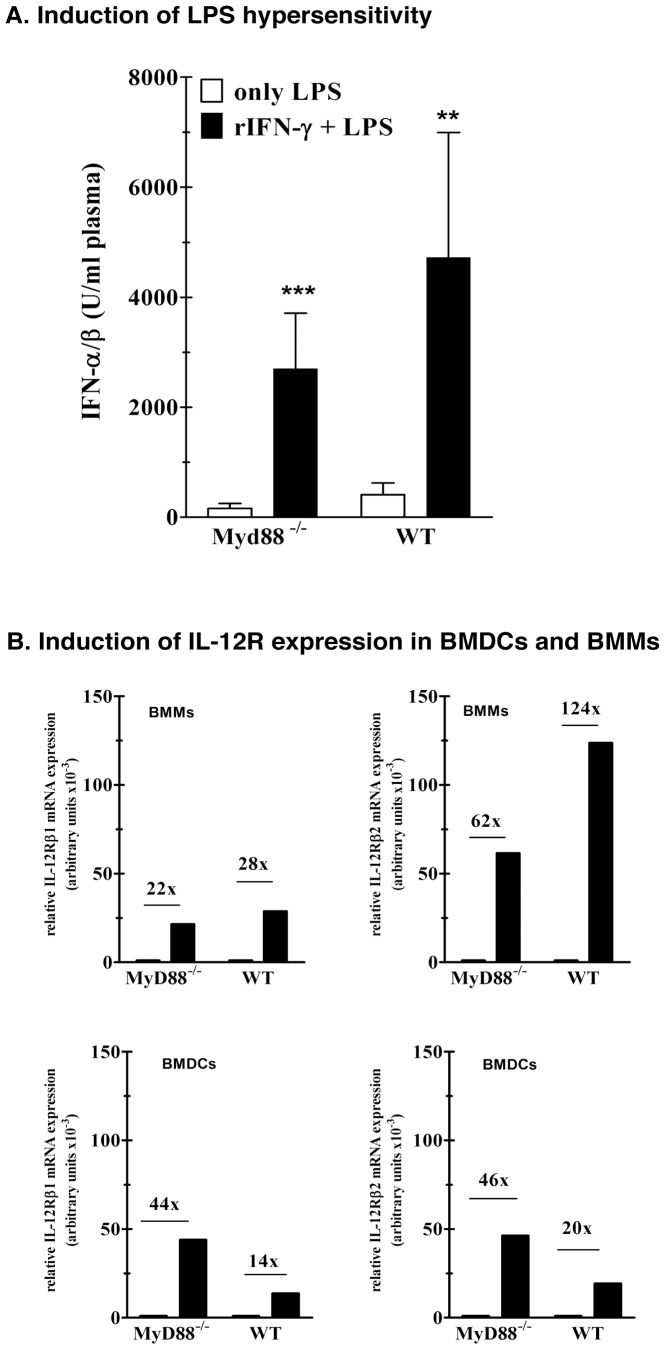
A. Induction of LPS hypersensitivity in mice pretreated with murine recombinant IFN-γ. **WT and MyD88^−/−^ mice were treated with recombinant IFN-γ (5000** **U/0.2** **ml) i.v.** or remained untreated (only LPS). Eight hours after pretreatment the animals were challenged with LPS *S.a.e.* (1 µg/g b.w.) i.v. and plasma was collected two hours later for determination of IFN-αβ. One representative experiment of three, with four to five mice is shown. **B**. Enhanced expression of IL-12Rβ1 and IL-12Rβ2 in MyD88^−/−^ and WT bone marrow derived macrophages and dendritic cells after stimulation with murine recombinant IFN**-**γ**.** 1.5×10^6^ bone marrow derived macrophages (BMM) or dendritic cells (BMDC) were stimulated with murine recombinant IFN-γ (rIFN-γ, 3000 U). After 24 hours, the cells were lysed in guanidinium-thiocyanate solution and lysates were used for RNA preparation as described in materials and methods. The mRNA levels were analyzed by quantitative real-time PCR. Each value is represented as a relative expression, which is evaluated after setting background expression in controls as arbitrary value  = 1. One representative experiment of two is shown.

Here, we compared the ability of IFN-γ to induce hyper-reactivity to LPS *in vivo* [5]. Wt and MyD88^−/−^ mice were treated with recombinant murine IFN-γ or vehicle (control) and the MyD88-independent IFN-αβ response to a LPS challenge was determined. In both types of mouse pretreatment with IFN-γ resulted in a 20-fold enhancement of the IFN-αβ response (Fig. 4A). We further investigated whether MyD88 deficiency affects the ability of IFN-γ to induce IL-12 receptor expression in macrophages and dendritic cells. The upregulation of IL-12R after *P. acnes*-priming on lymphocyte- and NK-cell depleted non-parenchymal liver cell population is a function of IFN-γ [6]. Using bone marrow derived macrophages (BMMs) and myeloid dendritic cells (BMDCs) we showed that IFN-γ strongly upregulates mRNA levels for IL-12Rβ1 and IL-12Rβ2 in both cell types, and that the absence of MyD88 does not impair this function (Fig. 4B). Thus, it is unlikely that impaired IFN-γ function could explain the absence of the early and late *P. acnes* effects in MyD88^−/−^ mice.

### Normal Development of Delayed *P. acnes* Effects in mice with Impaired Intracellular TLR Signaling due to UNC-93B Deficiency

To explain the requirement for MyD88, we next focused on the possible importance of intracellular TLRs, distinct from TLR9, in the late sensitization mechanism. For this purpose we used mice with a single point mutation in the ER-resident membrane protein UNC-93B (3d mice), which abolishes signaling *via* the intracellular TLRs, including TLR3, 7, 8, 9 and 13 [19]–[21]. As expected for animals with impaired TLR9 signaling, we observed no splenomegaly, LPS hypersensitivity (Figure S2), or intrahepatic granulomas (not shown) in the 3d mice on day 7 after priming. However, the UNC-93B deficiency did not abolish the delayed development of these characteristic *P. acnes* effects (Figure S2 and not shown). These results confirm the essential role of TLR9 in the early immune activation by *P. acnes* and suggest that intracellular UNC-93B-dependent TLRs do not have a critical function in the delayed activation.

### Normal Development of Delayed *P. acnes* Effects in mice with a Combined TLR2/9^−/−^, TLR4/9^−/−^ or TLR5/9^−/−^ Deficiency

TLR2, 4 and 5 are considered primarily cell surface receptors, but as shown at least for TLR2 and TLR4 [22]–[24], they can also localize and function intracellularly. *P. acnes*-primed TLR2^−/−^, TLR4^−/−^ [25] and TLR5^−/−^ (not shown) single deficient mice, like the respective wt mice, develop the characteristic early immune effects. To see whether combine defects in TLR2, 4 or 5 may abolish the TLR9-independent late sensitization by *P. acnes*, we generated TLR2/9^−/−^, TLR4/9^−/−^ and TLR5/9^−/−^ double deficient mice by crossbreeding. We primed all double deficient animals with *P. acnes* and challenged 21 days later with LPS (TLR2^−/−^ and TLR5^−/−^ mice) or the TLR2 ligand, synthetic lipopeptide (LP) FSL-1 (TLR4^−/−^ mice). Thereafter, TNF-α responses were determined. All double deficient mice exhibited strong late TNF-α hyper-responses (Figure S3) to the respective TLR ligands and developed splenomegaly and intrahepatic granulomas with a delay (not shown). Thus, the additional loss of function of the predominantly surface expressed TLRs 2, 4 and 5 does not impair the TLR9-independent late sensitization by *P.* *acnes*.

### Impairment of IL-1 or/and IL-18 Function Influences Neither TLR9-dependent nor -independent Immune Activation by *P. acnes*

MyD88 is also essential for signaling through the receptors for IL-1 and IL-18. To address the role of IL-1 and IL-18 in *P. acnes*-induced immune activation we generated TLR9^−/−^ mice containing additionally IL-1R or IL-18 deficiencies by backcrossing. Wt, IL-1R^−/−^, IL-18^−/−^, TLR9^−/−^, IL-1R/TLR9^−/−^ and IL-18/TLR9^−/−^ mice were challenged on day 7 or day 21 after *P. acnes* treatment with LPS and their TNF-α and IFN-γ responses, as well as those of the respective unprimed mice were determined. As shown in Figure S4A, the absence of IL-1 or IL-18 signaling in TLR9 competent mice impaired neither the early nor the late LPS sensitization. Furthermore, the absence of either cytokine signaling in TLR9^−/−^ mice did not impair the late sensitization (Figure S4B). In addition, we used Kineret to block IL-1 function in *P. acnes*-primed IL-18/TLR9^−/−^ double deficient mice. This concomitant loss of IL-1, IL-18 and TLR9 function also failed to abrogate the development of the delayed LPS sensitization (i.e. TNF-α or IFN-γ hyperresponses) after *P. acnes* treatment (Figure S4C).

### Impact of TLR2 and TLR4 Deficiency on the Delayed Development of *P. acnes* Immune Effects

**Figure 5:**
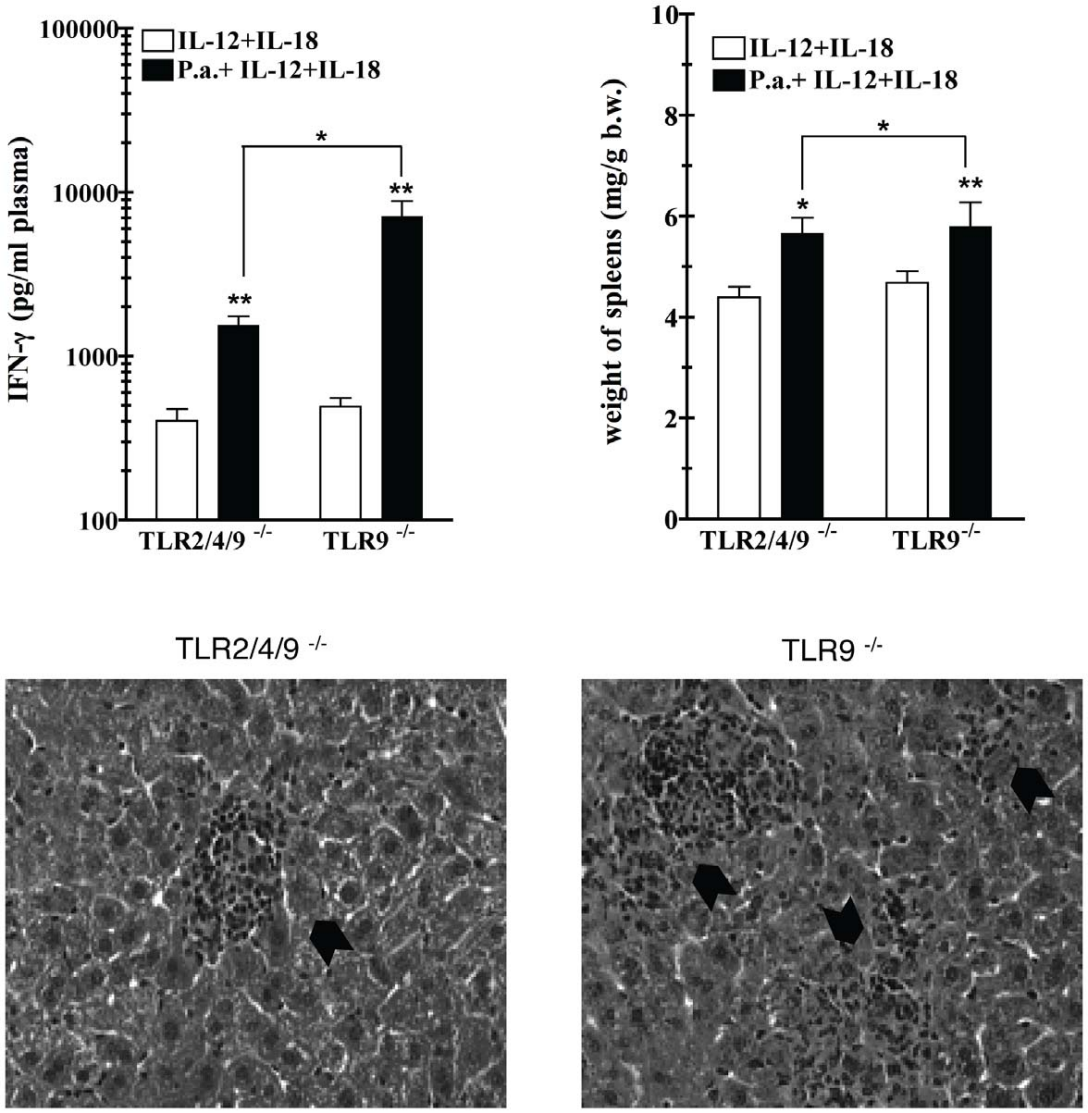
LPS sensitivity of *P. acnes* primed TLR2/TLR4/TLR9^−/−^ mice after. Groups of TLR9^−/−^ and TLR2/TLR4/TLR9^−/−^ mice were primed with heat-killed *P. acnes* (20 µg/g b.w.) i.v. or remained untreated. Each mouse was challenged 21 days after priming with a mixture of murine recombinant IL-12 (25 ng) and murine recombinant IL-18 (100 ng). Four hours after challenge, plasma was collected for determination of IFN-γ. Before challenge, no detectable IFN-γ was found in plasma of *P. acnes*-treated mice of either one of the groups (not shown). One representative experiment of two is shown. *:p-value<0.05 and **:p-value<0.01 as compared to only rIL-12+rIL-18-treated controls.

A growing number of reports show that TLRs subserve not only the recognition of microbial ligands (so called pathogen-associated molecular patterns, or PAMPs), but also of several endogenous ligands, designated damage-associated molecular patterns (DAMPs). In some cases, PAMPs and DAMPs may trigger more than one TLR receptor, as shown for degradation products of hyaluronic acid (HA) [26], [27], a ligand of TLR2 and TLR4. We showed previously that a double TLR2 and TLR4 deficiency does not impair the early sensitization to LPS in TLR9 competent mice [25]. In that study, strongly enhanced cytokine responses were found in TLR2/TLR4^−/−^ mice challenged 7 days after *P. acnes* priming with heat-killed gram-negative bacteria. To test whether a combined deficiency of TLR2 and 4 has a negative effect on the delayed development of *P. acnes* effects we generated TLR2/TLR4/TLR9 triple deficient mice by crossbreeding. We took advantage of the fact that *P. acnes*-primed mice become hypersensitive to IL-12, especially in combination with IL-18 [4], and used the IFN-γ response to a combined administration of the two cytokines as measure of late sensitization in *P. acnes*-treated mice. As shown in Fig. 5, the additional loss of both TLR2 and TLR4, contrary to that of either one alone (Figure S3), significantly reduced, but did not abolish the hypersensitivity on day 21 post priming in TLR9^−/−^ mice. Furthermore, it resulted in a significantly less pronounced splenomegaly and fewer and smaller granulomas in the liver (Fig. 5). These data strongly suggest that TLR2 and TLR4 contribute to the *P. acnes*-induced TLR9-independent late sensitization and in this function can substitute for each other.

## Discussion

Priming mice with heat-killed *P. acnes* is a well-established and widely used mouse model of immunomodulation by intracellular pathogens. We showed earlier and confirm here that the immunomodulatory effects of *P. acnes*, which are fully developed 7 days after priming, require the presence of functional TLR9. However, our study uncovers an unexpected alternative, TLR9-independent activation mechanism, developing with a marked delay of one to two weeks after *P. acnes* administration. Notably, the time required for complete elimination of the bacteria in mice (approximately 4 weeks) is longer in the absence of TLR9. The relative resistance of *P. acnes* to *in vivo* degradation is likely responsible for immunomodulation. Thus, only *P. acnes* strains or preparations which persist in the recipient mice for at least several days were shown to be stimulatory [8]. The extended duration of *P. acnes* persistence in TLR9^−/−^ deficient mice suggests an important role for TLR9 in bacterial degradation. However, the coincidence between the retarded degradation and the delayed appearance of immunomodulatory effects in TLR9^−/−^ mice opens the possibility that a certain degree of *P. acnes* degradation is necessary to initiate the development of these effects.

Interestingly, the alternative TLR9-independent activation mechanism leads to the same immune phenotype as the TLR9-dependent one, i.e. intrahepatic granuloma formation, splenomegaly, hypersensitivity to TLR ligands and endogenous mediators and enhanced resistance to infection. Since the adaptor MyD88 is essential for signaling through the majority of TLR receptors [17] and TLRs are important bacterial sensors, the involvement of MyD88 in the TLR9-independent late sensitization was not surprising. However, the unimpaired late development of the characteristic *P. acnes* effects in UNC-93B-deficient mice argues against participation of further intracellular TLRs such as TLR3, 7, 8, 11 and 13 [19]–[21]. Moreover, the additional absence of either TLR2, 4, or 5 individually did not impair the late immunomodulatory activity of *P. acnes* in TLR9^−/−^ mice. The role of TLR1 and TLR6 in *P. acnes* activation has not been experimentally excluded. However, so far known, these two TLRs heterodimerize with TLR2 to constitute functional receptors [28].

The crucial role for MyD88 in the late *P. acnes* sensitization may not only imply the requirement for a bacterial TLR sensor, but could also signalize the requirement for the IL-1-type cytokines. Microbial intracellular sensing *via* specific RIG-I like helicases (RLH) and the nucleotide binding domain (NBD) and leucine-rich repeat containing (NLRs) family members leads to proteolytic activation of IL-1β and IL-18, both signaling through MyD88-recruiting receptors. Although it is conceivable that peptidoglycan, released during *P. acnes* degradation in mice does activate NOD-1, NOD-2 [29], we excluded here a critical involvement of IL-1β and IL-18 in *P. acnes* sensitization by using respective knockout mice and the IL-1R antagonist Kineret. In accordance, mice deficient for the apoptosis-associated speck like protein (ASC), an adaptor essential for caspase-1-mediated IL-1β and IL-18 processing [30], exhibit normal immune responses to *P. acnes* priming (unpublished data).

Since IFN-γ plays a key role in both, the early [5] and late immunomodulatory response to *P. acnes* (this study) and in view of the importance of MyD88 in IFN-γ signaling (as reported by Sun et al. [18]), an impaired IFN-γ function would explain the complete absence of sensitization in *P. acnes*-treated MyD88^−/−^ mice. However, this seems unlikely, since upregulation of IL-12R in macrophages and dendritic cells and induction of LPS hypersensitivity in mice, two activities of IFNγ relevant for *P. acnes* sensitization [5], [6], remained unimpaired in the absence of MyD88.

Our study shows that deficiency of a single of the known MyD88-dependent TLR receptors does not inhibit the late, TLR9-independent immunomodulatory response to *P. acnes*. However, important physiological functions are often multiple safeguarded. For example, the TNF-α response, which is essential for infection control, is triggered by several microbial PAMPs of a sole pathogen through their specific TLRs. Interestingly in some cases one TLR ligand activates more than one receptor. This was documented for degradation products of hyaluronic acid (HA) generated in reaction to tissue damage [31], [32]. These products, depending on molecular size, activate both TLR2 and TLR4 [26], [27]. Importantly, experiments carried out in mice suggest strongly that endogenous DAMPs activate both TLR2 and TLR4 and play a decisive role in the induction of murine allergic contact hypersensitivity [33] and in protection of lung injury in mice injected with bleomycin [34]. Previously, we excluded a role for TLR2 and TLR4 in the early, TLR9-dependent *P. acnes* sensitization to LPS and lipopeptide [25]. However, this study demonstrates a significant negative influence of the combined TLR2/4 deficiency on the late sensitization. Although TLR2 has been identified as a sensor of *P. acnes* [35], a similar property for TLR4 was not seen in our studies (unpublished data), nor reported elsewhere. Therefore, a possible involvement of DAMPs induced by the unusually long persistence of bacteria in host cells may be envisaged, which suggests germ-free mice as a future experimental model.

At present we cannot determine whether the redundant action of multiple Myd88-dependent receptors, or an as-yet-unknown MyD88 function, together with the activity of TLR2 and TLR4 drives the late immunomodulation in *P. acnes*-primed TLR9^−/−^ mice. On the whole, our study shows that the immunomodulation by *P. acnes*, which is primarily TLR9-dependent, proceeds in the absence of TLR9 with a delay by an alternative, MyD88-dependent mechanism. At least two players involved in this mechanism, TLR2 and TLR4, seem to fulfill a redundant role. Most likely, an IFN-γ-dependent and TLR9-independent mechanism also participate in immune responses to other microbes and/or DAMPs generated by sterile injury, hypothesis which warrant further investigation. Its elucidation will contribute to new strategies for diagnosis and a more specific treatment of numerous inflammatory diseases.

## Materials and Methods

### Materials

A highly pure preparation of *Salmonella abortus equi* LPS in the uniform triethylamine salt form and heat-killed *P. acnes* were prepared as described previously [5], [36]. FSL-1 was purchased from EMC microcollections (Tübingen, Germany), recombinant murine IFN-γ and IL-12 from NatuTec (Frankfurt a.M., Germany), murine IL-18 from MBL laboratories (Nagoya, Japan) and IL-1 receptor antagonist Kineret (Amgen Europe B.V.) was kindly provided by S. Martin (University of Freiburg, Germany). Antiserum to *P. acnes* was prepared by immunizing rabbits sub-cutaneously with heat-killed *P. acnes,* as described earlier [37]. Serum from non-immunized rabbits served as control. The antiserum and control serum were absorbed with washed murine splenocytes to remove antibodies directed to mouse tissue.

### Animals

Wt (C57BL/10ScSn, C57BL/6, 129Sv/Pas) and all deficient mice were bred under specific pathogen-free (SPF) conditions in the animal facilities of the Max-Planck-Institute of Immunbiology and Epigenetics. C57BL/10ScN mouse contains a spontaneous entire deletion of the entire *Tlr4* locus [38]. IL-12Rβ2-deficient mice (spontaneous mutation; C57BL/10 background) express a non-functional IL-12R [39]. Breeding pairs of UNC93B^−/−^ [21] and TRIF^−/−^ mice [40] generated by random germ line mutagenesis, TLR9^−/−^ [41], IL-1R^−/−^ mice [42] and MyD88^−/−^ [43] knockout mice were kindly provided by B. Beutler (The Scripps Research Institute, La Jolla), H. Wagner (Institute of Medical Microbiology, Immunology, and Hygiene, Technische Universität, Munich, Germany), H. Bluethmann (Transgenic animal models, Hoffmann-La Roche, Basel), M. Kopf (TH Zürich) and R. Landmann (Department Forschung, Kantonspital Basel). All single deficient knockout mice were backcrossed 7–11 times to a C57BL/6 or C57BL/10 background. IFN-γ^−/−^ mice were bred on a 129Sv/Ev background [44]. All double deficient mice (TLR2/TLR9^−/−^, TLR4/TLR9^−/−^, TLR5/TLR9^−/−^, IL-1R/TLR9^−/−^ and IL-18/TLR9^−/−^) were generated by crossbreeding respective single deficient strains and backcrossing the first generation twice to the TLR9^−/−^ mice of the respective background (BL/10 or BL/6). The triple deficient TLR2/TLR4/TLR9^−/−^ (BL/10) mice were obtained after crossbreeding TLR9^−/−^ with TLR2/TLR4^−/−^ mice and backcrossing the first generation twice to the TLR9^−/−^ strain.

For priming, both male and female mice, 7–10 weeks of age received 20 µg *P. acnes*/g body weight (b.w.), intravenously (i.v.). Control mice remained untreated. To inhibit IL-1 function, mice were treated intraperitoneally (i.p.) with 200 µg Kineret/mouse, 1 day prior to and every other day after *P. acnes* treatment until 1 day prior challenge. For cytokines induction *P. acnes*-treated and untreated control mice were challenged at the indicated time point after priming with either LPS (0.01 µg/g b.w.) or FSL-1 (0.5 µg/g b.w.), or a combination of rIL-12 and rIL-18 (25 ng and 100 ng/mouse, respectively), i.v. Heparinized blood was collected for TNF-α, IFN-αβ and IFN-γ measurements, 1 h, 2 h and 4 h after challenge, respectively.

All experimental procedures were in accordance with institutional, state and federal guidelines on animal welfare.

### Infection

A highly virulent strain of serovar Typhimurium C5 was grown and the exact numbers of bacteria determined as described previously [12]. For infection, 200 CFU of bacteria suspended in 0.2 ml of PBS were administered i.v. into the lateral tail vein of mice. Determination of viable bacteria in the liver was carried out 4 days after infection, as described in [12].

### Cell Culture

Bone marrow-derived macrophages (BMMs) were generated from bone marrow precursor cells in the presence of L-cell-conditioned medium in hydrophobic Teflon bags as described previously [45]. After 10 days of culture the cells were washed and resuspended at a concentration of 5×10^5^/ml in a serum free, high-glucose formulation of Dulbecco’s modified Eagle medium (DMEM) for further use. GM-CSF induced bone marrow-derived dendritic cells (BMDCs) were generated as described previously [46]. Mature cells were washed and resuspended at a concentration of 10^6^ cells/ml serum-free RPMI for further use.

### Cytokine Determination

TNF-α was measured in a bioassay cytotoxicity test using a TNF-sensitive L929 cell line of fibroblasts [47] (kindly provided by L. Old, Memorial Sloan-Kettering Cancer Center, New York, NY) in the presence of actinomycin D as described previously [5]. The detection limit of the assay was 32 pg TNF-α/ml plasma. IFN-αβ levels were measured using a L929 cell line transfected with an interferon-sensitive (ISRE) luciferase construct [48]. The detection limit of the assay was 1 unit/ml. IFN-γ was measured using the OptIEA ELISA kit from BD Biosciences (San Diego, CA) according to the manufacturer’s instructions. The detection limit of the assay was 50 pg IFN-γ/ml plasma.

### Histopathology and Immunoperoxidase Staining Procedure

Liver, spleen and kidneys of sacrificed mice were fixed in 4% buffered formaldehyde and paraffin-embedded using routine protocols. Horizontal organ sections (5 µm thick) were stained using H&E. To detect *P. acnes*, 5 µm thick horizontal organ sections were stained with anti-*P. acnes* or control serum using the avidin-biotin peroxidase (ABC Method), as described previously [49]. Cell nuclei were counterstained with hematoxylin (blue). Stained sections were viewed by light microscopy and pictures were taken from representative areas of the organ. Brownish color indicates the presence of *P. acnes* antigen.

### RT-PCR and Real-time RT-PCR

Total RNA was isolated from cells or freshly removed organs using a guanidinium isothiocyanate-phenol-chloroform-isoamyl alcohol procedure [50], [51] and treated with RNase free DNase I (Fermentas, St Leon-Rot, Germany) to enhance the purity of the extracted RNA. The RNA concentration was determined by absorbance at 260 nm. RNA (1 µg) was reverse transcribed with Moloney Murine Leukemia Virus reverse transcriptase (M-MuLV-RT) and oligo-p(dT) primers (Expand reverse transcriptase kit; Roche, Basel, Switzerland) according to the manufacturer’s instructions. RT-PCR was performed as described previously [9]. Real-time PCR quantification of RNA expression was performed using the LightCycler II system (Roche) and the Quantitect SYBR Green PCR Kit (Qiagen) according to the instructions of the manufacturers. Arbitrary units of relative expression were generated by dividing the value obtained for mRNAs under test by the value of β-actin and multiplying the result by 1000. The following primer pairs were used: β-Actin: sense, 5′-TGGAATCCTGTGGCATCCATGAAAC; antisense, 5′-TAAAACGCAGCTC AGTAACAGTCCG (348 bp product size). IL-12Rβ1: sense, 5′- AGGCCGCCCCTGAGTGCGCC; antisense, 5′- GGCGGTCCCGGCTCCGCAGT (230 bp product size) and IL-12Rβ2: sense, 5′- TTCTTCTTCACTTCCGCATA; antisense, 5′- CAGATCTCGCAGGTCATATT (210 bp product size). An annealing temperature of 58°C was used for all primer pairs. β-actin expression was used to normalize cDNA levels. The PCR products were analyzed by melting curve and agarose gel electrophoresis analyses to ascertain the specificity of the amplification.

### Data Analysis and Statistics

Data were analyzed using Prism GraphPad 4.0 software. Data in figures are presented as mean, error bars show SEM. Statistical analysis was performed with the unpaired t-test (*:p<0.05; **:p<0.01; ***:p<0.001).

## Supporting Information

Figure S1 **LPS sensitivity of** ***P. acnes*** **primed IFN-γR^−/−^, TNF-α^−/−^** **and IL-12Rβ2^−/−^ mice.** Groups of four to five mice were treated with heat-killed *P. acnes* (20 µg/g b.w.) i.v. or remained untreated (only LPS). 7 and 21 days after priming the animals were challenged with LPS *S.a.e.* (0.01 µg/g b.w.) i.v. and plasma was collected one hour later for determination of TNF-α. One representative experiment of two is shown. **:p-value<0.01 and ***:p-value<0.001.
(PPT)

Figure S2 **Delayed development of splenomegaly and LPS hypersensitivity in 3d mice after** ***P. acnes*** **.** Groups of 4 to 5 mice were treated with heat-killed *P. acnes* (20 µg/g b.w.) i.v. or remained untreated (only LPS). The animals were challenged after 7 and 21 days with LPS *S.a.e.* (0.01 µg/g b.w.) i.v. One hour and 4 h later, plasma was collected for determination of TNF-α and IFN-γ, respectively. Before challenge with LPS no detectable TNF-α or IFN-γ was found in plasma of *P. acnes*-treated mice of either one of the groups (not shown). One representative experiment of three is shown. *:p-value<0.05, **:p-value<0.01 and ***:p-value<0.001.
(PPT)

Figure S3 **LPS sensitivity of** ***P. acnes*** **primed TLR2/TLR9^−/−^, TLR4/TLR9^−/−^ and TLR5/TLR9^−/−^ mice.** Groups of 5 mice were primed with heat-killed *P. acnes* (20 µg/g b.w.) i.v. or remained untreated (only LPS, only FSL-1). After 21 days, unprimed and primed TLR2/TLR9^−/−^ and TLR5/TLR9^−/−^ mice were challenged with LPS *S.a.e* (0.01 µg/g b.w.), while TLR4/TLR9^−/−^ mice were challenged with FSL-1 (0.5 µg/g b.w.). As control, TLR9^−/−^ mice were challenged with either LPS *S.a.e* or FSL-1. One hour and 4 h after challenge, plasma was collected for determination of TNF-α and IFN-γ, respectively. Before challenge, no detectable TNF-α or IFN-γ was found in plasma of *P. acnes*-treated mice of either one of the groups (not shown). One representative experiment of two is shown. *:p-value<0.05 and **:p-value<0.01 as compared to only LPS or to only FSL-1 treated controls.
(PPT)

Figure S4 **A. and B. Delayed development of LPS hypersensitivity in IL-1R^−/−^, IL-18^−/−^, TLR9/IL-1R^−/−^ and TLR9/IL-18^−/−^ mice after** ***P. acnes*** **priming.** Groups of 4–5 mice were treated with heat-killed *P. acnes* (20 µg/g b.w.) i.v. or remained untreated (only LPS). 7 and/or 21 days later, the animals were challenged with LPS *S.a.e.* (0.01 µg/g b.w.) i.v. One hour and 4 h later, plasma was collected for determination of TNF-α and IFN-γ, respectively. Before challenge with LPS no detectable TNF-α or IFN-γ was found in plasma of *P. acnes*-treated mice of either one of the groups (not shown). **C. LPS sensitivity of TLR9/IL-18^−/−^ mice with impaired IL-1 production.** Groups of TLR9/IL-18^−/−^ mice were primed with heat-killed *P. acnes* (20 µg/g b.w.) i.v. or remained untreated (LPS). One day prior and every other day after priming, the animals were treated with kineret (200 µg/mouse) or vehicle control (PBS) i.p., to antagonize the IL-1 activity. At day 14 after priming, the mice were challenged with LPS *S.a.e.* (0.01 µg/g b.w.) i.v. One hour and 4 h later, plasma was collected for determination of TNF-α and IFN-γ, respectively. Spleens were removed, weighted and spontaneous IL-1β production was determined from the collected supernatant of lysed splenocytes. One representative experiment of three is shown. *:p-value<0.05, **:p-value<0.01 and ***:p-value<0.001.
(PPT)
