# Supplementary figures and images for: TLR9-Dependent and Independent Pathways Drive Activation of the Immune System by Propionibacterium Acnes
Source: PLoS One. 2012 Jun 22;7(6):e39155. doi: 10.1371/journal.pone.0039155 (PMC3382180; doi:10.1371/journal.pone.0039155)

## Slide 1
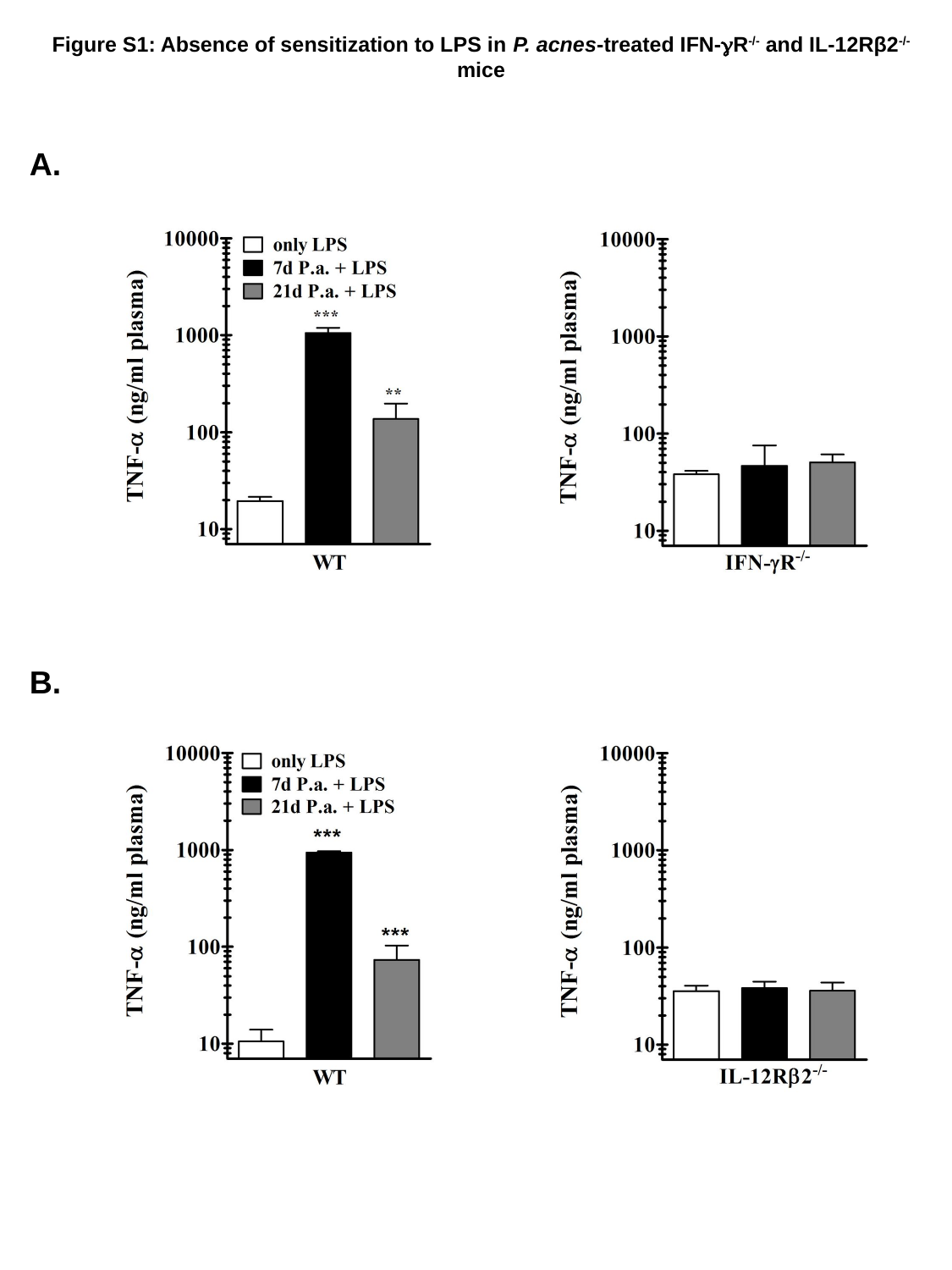

# Figure S1: Absence of sensitization to LPS in P. acnes-treated IFN-γR-/- and IL-12Rβ2-/- mice
A.
B.

Supplement: Figure S1 — LPS sensitivity of P. acnes primed IFN-γR−/−, TNF-α−/− and IL-12Rβ2−/− mice. Groups of four to five mice were treated with heat-killed P. acnes (20 µg/g b.w.) i.v. or remained untreated (only LPS). 7 and 21 days after priming the animals were challenged with LPS S.a.e. (0.01 µg/g b.w.) i.v. and plasma was collected one hour later for determination of TNF-α. One representative experiment of two is shown. **:p-value<0.01 and ***:p-value<0.001. (PPT) [file pone.0039155.s001.ppt]
