# Supplementary material for: TLR9-Dependent and Independent Pathways Drive Activation of the Immune System by Propionibacterium Acnes
Source: PLoS One. 2012 Jun 22;7(6):e39155. doi: 10.1371/journal.pone.0039155 (PMC3382180; doi:10.1371/journal.pone.0039155)
Supplement: Figure S2 — Delayed development of splenomegaly and LPS hypersensitivity in 3d mice after P. acnes . Groups of 4 to 5 mice were treated with heat-killed P. acnes (20 µg/g b.w.) i.v. or remained untreated (only LPS). The animals were challenged after 7 and 21 days with LPS S.a.e. (0.01 µg/g b.w.) i.v. One hour and 4 h later, plasma was collected for determination of TNF-α and IFN-γ, respectively. Before challenge with LPS no detectable TNF-α or IFN-γ was found in plasma of P. acnes-treated mice of either one of the groups (not shown). One representative experiment of three is shown. *:p-value<0.05, **:p-value<0.01 and ***:p-value<0.001. (PPT) [file pone.0039155.s002.ppt]

## Slide 1
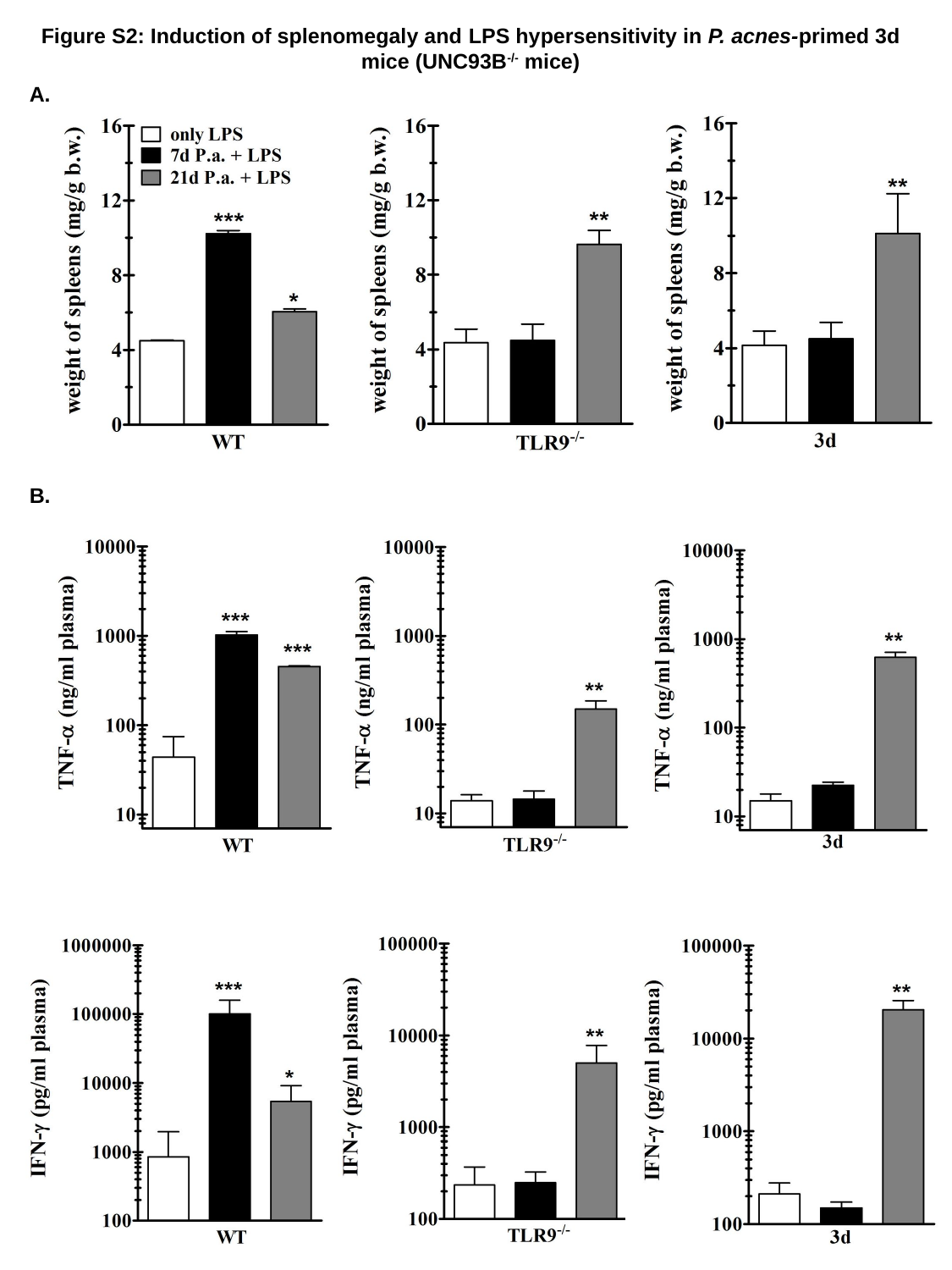

# Figure S2: Induction of splenomegaly and LPS hypersensitivity in P. acnes-primed 3d mice (UNC93B-/- mice)
A.
B.
