# Supplementary material for: TLR9-Dependent and Independent Pathways Drive Activation of the Immune System by Propionibacterium Acnes
Source: PLoS One. 2012 Jun 22;7(6):e39155. doi: 10.1371/journal.pone.0039155 (PMC3382180; doi:10.1371/journal.pone.0039155)
Supplement: Figure S3 — LPS sensitivity of P. acnes primed TLR2/TLR9−/−, TLR4/TLR9−/− and TLR5/TLR9−/− mice. Groups of 5 mice were primed with heat-killed P. acnes (20 µg/g b.w.) i.v. or remained untreated (only LPS, only FSL-1). After 21 days, unprimed and primed TLR2/TLR9−/− and TLR5/TLR9−/− mice were challenged with LPS S.a.e (0.01 µg/g b.w.), while TLR4/TLR9−/− mice were challenged with FSL-1 (0.5 µg/g b.w.). As control, TLR9−/− mice were challenged with either LPS S.a.e or FSL-1. One hour and 4 h after challenge, plasma was collected for determination of TNF-α and IFN-γ, respectively. Before challenge, no detectable TNF-α or IFN-γ was found in plasma of P. acnes-treated mice of either one of the groups (not shown). One representative experiment of two is shown. *:p-value<0.05 and **:p-value<0.01 as compared to only LPS or to only FSL-1 treated controls. (PPT) [file pone.0039155.s003.ppt]

## Slide 1
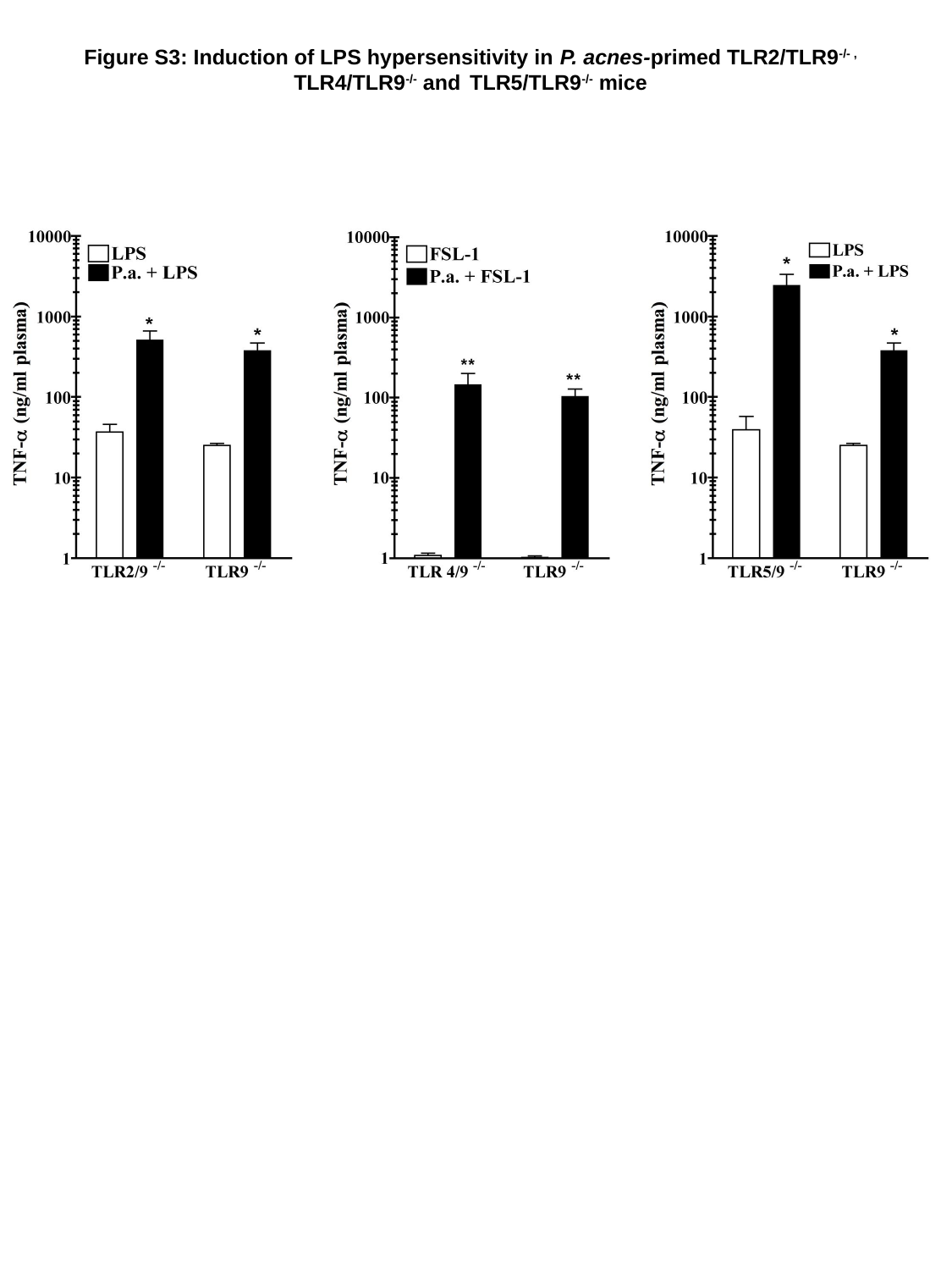

# Figure S3: Induction of LPS hypersensitivity in P. acnes-primed TLR2/TLR9-/- , TLR4/TLR9-/- and TLR5/TLR9-/- mice
