# Supplementary material for: TLR9-Dependent and Independent Pathways Drive Activation of the Immune System by Propionibacterium Acnes
Source: PLoS One. 2012 Jun 22;7(6):e39155. doi: 10.1371/journal.pone.0039155 (PMC3382180; doi:10.1371/journal.pone.0039155)
Supplement: Figure S4 — A. and B. Delayed development of LPS hypersensitivity in IL-1R−/−, IL-18−/−, TLR9/IL-1R−/− and TLR9/IL-18−/− mice after P. acnes priming. Groups of 4–5 mice were treated with heat-killed P. acnes (20 µg/g b.w.) i.v. or remained untreated (only LPS). 7 and/or 21 days later, the animals were challenged with LPS S.a.e. (0.01 µg/g b.w.) i.v. One hour and 4 h later, plasma was collected for determination of TNF-α and IFN-γ, respectively. Before challenge with LPS no detectable TNF-α or IFN-γ was found in plasma of P. acnes-treated mice of either one of the groups (not shown). C. LPS sensitivity of TLR9/IL-18−/− mice with impaired IL-1 production. Groups of TLR9/IL-18−/− mice were primed with heat-killed P. acnes (20 µg/g b.w.) i.v. or remained untreated (LPS). One day prior and every other day after priming, the animals were treated with kineret (200 µg/mouse) or vehicle control (PBS) i.p., to antagonize the IL-1 activity. At day 14 after priming, the mice were challenged with LPS S.a.e. (0.01 µg/g b.w.) i.v. One hour and 4 h later, plasma was collected for determination of TNF-α and IFN-γ, respectively. Spleens were removed, weighted and spontaneous IL-1β production was determined from the collected supernatant of lysed splenocytes. One representative experiment of three is shown. *:p-value<0.05, **:p-value<0.01 and ***:p-value<0.001. (PPT) [file pone.0039155.s004.ppt]

## Slide 1
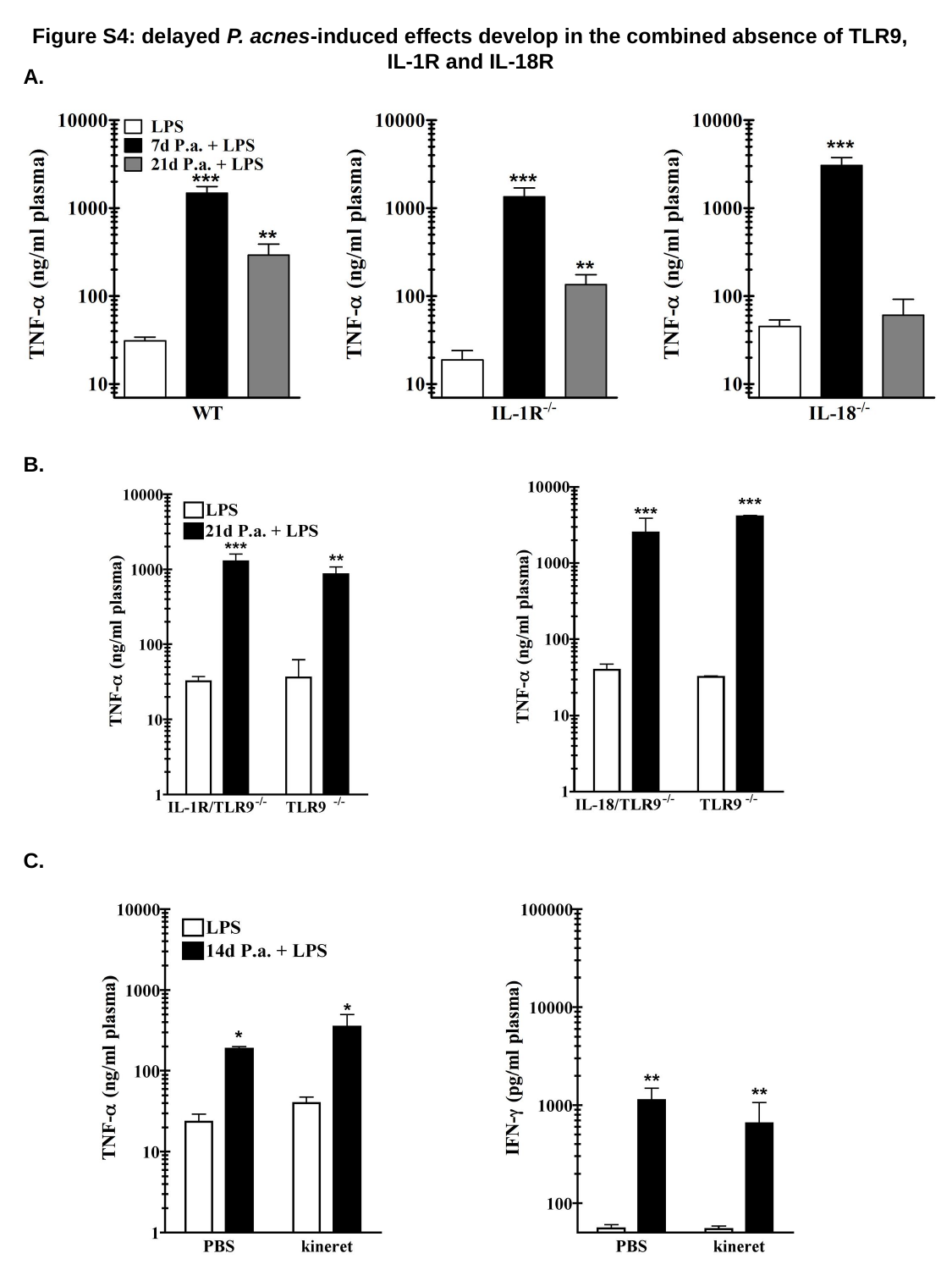

# Figure S4: delayed P. acnes-induced effects develop in the combined absence of TLR9, IL-1R and IL-18R
A.
B.
C.
